# A Case of Displaced Cricoid Cartilage Fracture Successfully Treated With Non-Surgical Intervention

**DOI:** 10.7759/cureus.83281

**Published:** 2025-04-30

**Authors:** Raiki Tokutsu, Takero Terayama, Tatsunori Nagamura, Takashi Nishiyama, Kosuke Hatanaka

**Affiliations:** 1 Department of Emergency Medicine, Self-Defense Forces Central Hospital, Tokyo, JPN; 2 Department of Emergency Medicine, National Defense Medical College, Tokorozawa, JPN; 3 Emergency and Critical Care Center, Okinawa Prefecture Chubu Hospital, Uruma, JPN

**Keywords:** blunt cervical trauma, conservative treatment, cricoid cartilage, fiberoptic laryngoscopy, tracheotomy

## Abstract

Laryngeal trauma, particularly cricoid fractures, is rare but potentially life-threatening and often requires invasive airway management. However, factors contributing to successful conservative treatment remain unclear. We report the case of a 40-year-old woman diagnosed with a displaced cricoid cartilage fracture following a motorcycle accident. Despite the risk of upper airway obstruction due to a massive hematoma observed on fiberoptic laryngoscopy and a displaced cricoid cartilage fracture, the patient was successfully managed conservatively without tracheotomy or endotracheal intubation through frequent airway follow-up. The patient was discharged on day 4, and no complications occurred as of eight weeks after discharge. Conservative management may be a viable option for laryngeal injuries, particularly when clinical examination findings, indicating no airway urgency, are prioritized over imaging techniques such as computed tomography or laryngoscopy.

## Introduction

Laryngeal trauma is a rare injury, occurring in approximately 1% of blunt trauma cases, with cricoid injuries accounting for approximately 50% of laryngeal trauma [1]. In such cases, early identification, prompt evaluation, and judicious management are essential to prevent serious complications and improve clinical outcomes, such as mortality. For the management of unstable airways, tracheotomy is preferred over endotracheal intubation [2], even though it has been reported that careful endotracheal intubation could successfully establish a secure airway [3]. However, the factors that contribute to avoiding invasive airway management, such as tracheotomy or endotracheal intubation, remain unclear. Herein, we report a successful case of an isolated, displaced cricoid fracture treated conservatively with repeated fiberoptic laryngoscopies at short intervals.

## Case presentation

A 40-year-old woman was admitted to the emergency department (ED) following a motorcycle accident and presented with blunt cervical trauma. She fell to the ground and sustained anterior neck bruising while mounting her motorcycle. Her chief complaints were hoarseness and odynophagia, without neck pain or respiratory distress.

On examination, her consciousness was clear (Glasgow Coma Scale score: E4V5M6), with a blood pressure of 112/68 mmHg, pulse rate of 55 beats per minute, and respiratory rate of 18 breaths per minute. Her arterial oxygen saturation was 100%. Physical examination in the ED revealed a bruise around the cricoid cartilage; however, no subcutaneous emphysema, lacerations, or severe airway stenosis symptoms, such as wheezing, tachypnea, or stridor, were observed.

**Figure 1:**
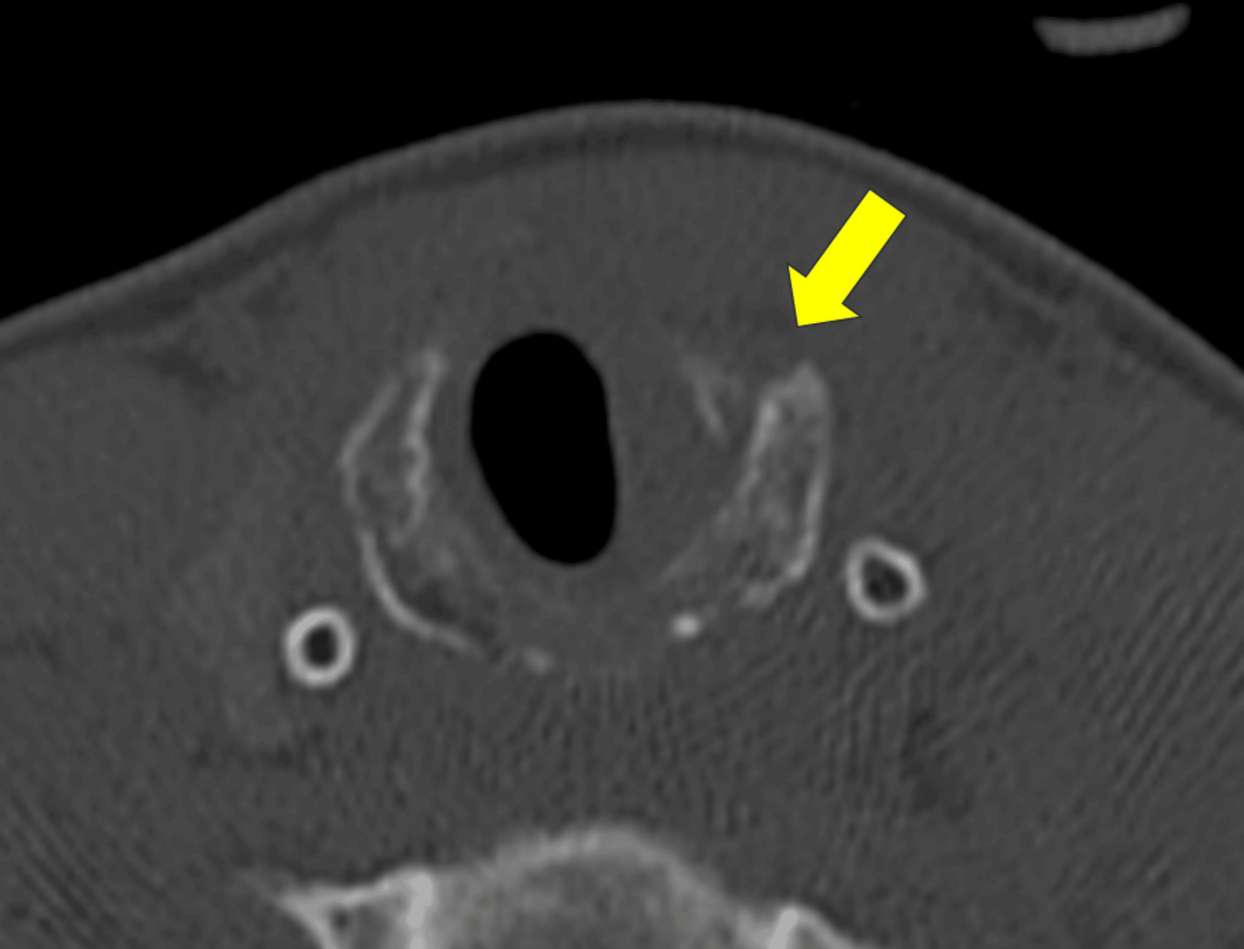
CT of the neck at the emergency department demonstrating a displaced fracture of the left anterior arch of the cricoid cartilage (arrow) with a hematoma.

**Figure 2:**
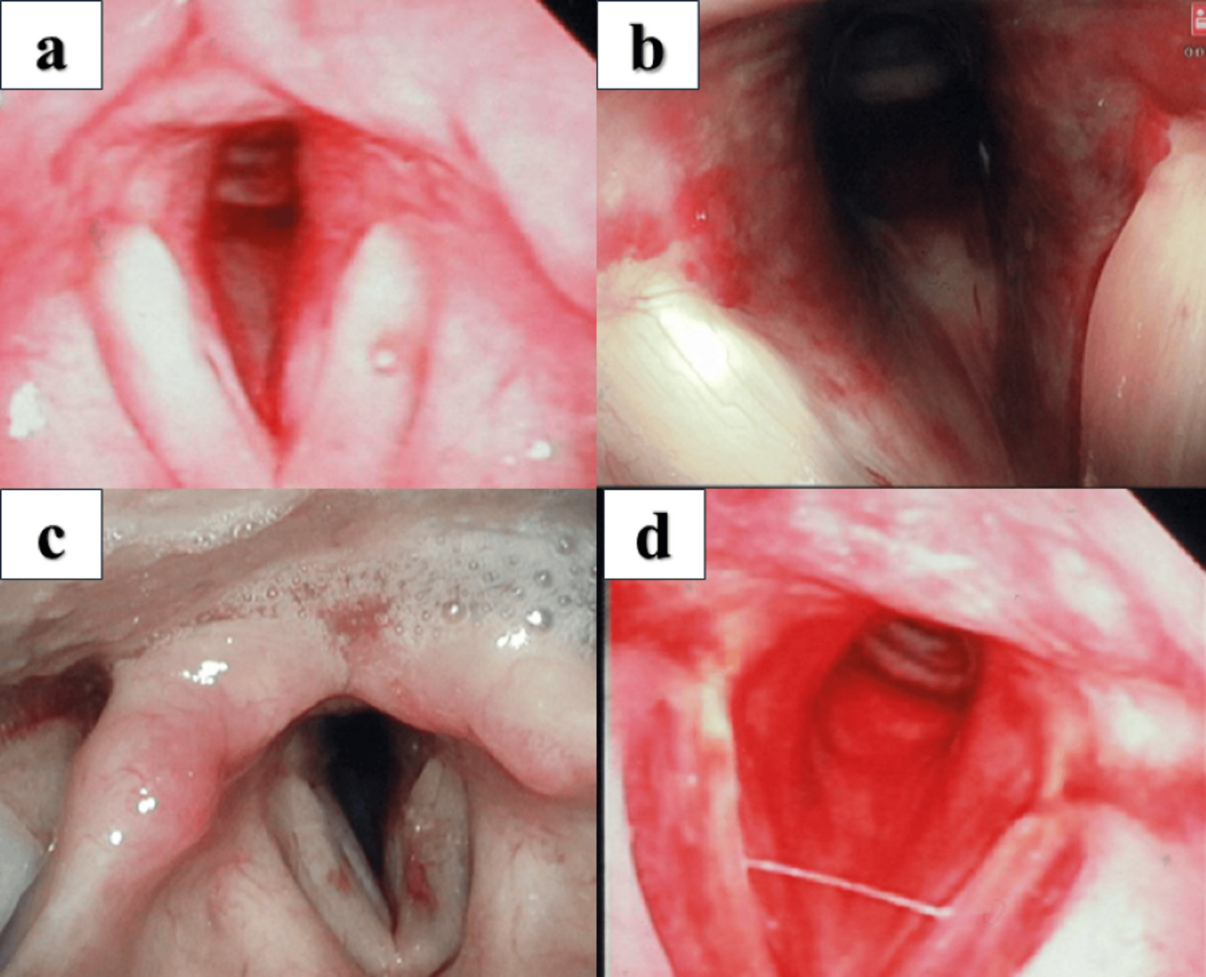
Fiberoptic laryngoscopy findings (a) Initial examination at admission showing a massive hematoma obstructing the airway. (b) Follow-up examination one hour later demonstrating no progressive swelling. (c) Follow-up examination 24 hours after admission confirming the absence of progressive swelling. (d) Examination on day 5 revealing complete resolution of the hematoma.

Computed tomography (CT) of the neck revealed a displaced fracture of the left anterior arch of the cricoid cartilage (Figure 1). Fiberoptic laryngoscopy revealed a massive hematoma obstructing approximately 40% of the airway; however, there was no evidence of left vocal cord immobility, exposed cartilage, or mucosal tears (Figure 2A). 

Conservative treatment without invasive airway management was initiated with intravenous methylprednisolone (500 mg/day) and frequent follow-up with fiberoptic laryngoscopy. Follow-up examinations performed at one and 24 hours after the initial assessment revealed no progressive swelling (Figures 2B, 2C). Therefore, although tracheotomy and endotracheal intubation were considered, they were ultimately deferred, and the patient was successfully managed with continued conservative treatment. The patient was discharged on day 4. At the follow-up examination on day 5, the hematoma had resolved (Figure 2D), and the hoarseness had disappeared by week 8.

## Discussion

This was a successful case of conservative treatment in a patient with a displaced cricoid cartilage fracture and airway stenosis. The case suggests that noninvasive management of displaced cricoid cartilage fractures as the initial treatment is feasible under specific circumstances.

Cricoid cartilage fractures are uncommon injuries caused by blunt neck trauma. Focal subglottic hematomas are significantly associated with multisite fractures involving the cricoid cartilage [1,4]. The classification system proposed by Fuhrman and Schaefer is widely referenced and divides blunt laryngeal trauma into five groups based on symptoms and findings on CT or fiberoptic laryngoscopy: Group 1 (minor endolaryngeal hematoma or laceration without detectable fractures), Group 2 (edema, hematoma, minor mucosal disruption without exposed cartilage, and non-displaced fractures noted on computed tomography), Group 3 (massive edema, mucosal tears, exposed cartilage, cord immobility, and displaced fractures), Group 4 (same as Group 3 with more than two fracture lines or massive trauma to the laryngeal mucosa), and Group 5 (complete laryngotracheal separation)[1,2]. The management protocol by Harris and Tobin (HT protocol) is often referenced, demonstrating three levels of treatment: Level I (observation with/without direct laryngoscopy), Level II (direct laryngoscopy and open surgical repair), and Level III (direct laryngoscopy and open surgical repair with stent placement) [5]. Butler et al. reported normal vocal cord mobility, minor hematoma formation, minimal mucosal trauma, no cartilage exposure, and no multiple or displaced fractures in successful cases of conservative treatment [6].

Early intervention with invasive airway management is associated with better voice and airway function [6,7]. Tracheostomy performed within 24 hours after injury is also reported to be associated with a shorter length of intensive care unit stay and hospital stay [8]. However, attempting endotracheal intubation in patients with laryngeal injuries can also result in critical airway complications such as fistula, further injury to the larynx, and mucosal disruption. In cases of laryngeal trauma accompanied by tracheal injury, the failure rate of endotracheal intubation has been reported to be as high as 76% [9]. Tracheotomy under local anesthesia is recommended for secure airway control, although it carries the risk of massive bleeding and failure [10].

Thus, careful decision-making regarding invasive airway management is required for patients with laryngeal trauma, considering its risks and benefits. Secure airway management is one of the most controversial issues. Three successful cases of conservative treatment have been reported, where tracheostomy was recommended based on the HT protocol [11,12]. These cases indicate that isolated low-energy blunt trauma, clinically stable conditions, absence of airway emergencies such as subcutaneous emphysema or tachypnea, and availability of frequent follow-up laryngoscopy play an important role in avoiding invasive airway management.

In this case, we could avoid invasive airway management, although secure airway management should have been recommended because of the displaced cricoid cartilage and massive hematoma obstructing approximately 40% of the airway. Our case was more severe than previously reported cases based on the findings from CT and fiberoptic laryngoscopy. There is no established evidence regarding the degree of airway stenosis that warrants endotracheal intubation. Our case suggests that prioritizing physical examination findings over CT or laryngoscopy findings in ambiguous cases of secure airway management can help avoid unnecessary invasive airway procedures. It is essential to confirm the absence of active bleeding around the injury site and prepare for immediate and secure airway management.

The characteristics of reported cases in which noninvasive management was successful, including the present case, are given in Table 1.

**Table 1:** Characteristics of successful cases of conservative treatment of laryngeal trauma. Note: '+' indicates presence of symptoms, '-' indicates absence of symptoms. ‡ Time from the first laryngoscopy examination

Characteristics	Falcone et al., 2017 [11]	Falcone et al., 2017 [11]	Oh et al., 2007 12］	Present case
Age (year)	20s	30s	32	40
Sex	Male	Male	Female	Female
Mechanism of injury	Fell off a skateboard	Accidentally struck by a barbell	Strangled by a person	Fell off a bicycle
Trauma diagnosis	Isolated cricoid cartilage fracture	Isolated cricoid cartilage fracture	Isolated cricoid cartilage fracture	Isolated cricoid cartilage fracture
Classification of laryngeal injury	Group 3	Group 3	Group 3	Group 3
Classification of injury management	Level Ⅱ	Level Ⅱ	Level Ⅱ	Level Ⅱ
Symptoms related to upper airway				
Hoarseness	+	+	+	+
Neck pain	+	+	+	-
Dyspnea	-	-	+	-
Stridor	-	-	+	-
Odynophagia	-	+	-	+
Hemoptysis	+	-	-	-
Timing of laryngoscopy^‡^ (hour)	0, 48	0, 24	0, 6, 9, 12	0, 1, 24, 120
Clinical outcome				
Mortality	Survive	Survive	Survive	Survive
Voice function	Not available	Not available	Recover	Recover

## Conclusions

This report highlights the successful conservative treatment of a patient with a laryngeal injury, preserving vocal cord function and ensuring a favorable prognosis. Generally, treatment should be guided by the Butler classification and the HT protocol. In ambiguous cases regarding invasive airway management as the initial treatment, prioritizing clinical physical examination findings over those of CT or laryngoscopy can help avoid unnecessary invasive airway procedures. Further studies with larger sample sizes are required to investigate the key factors for avoiding invasive management.
